# Case Report: Pseudoprogression of pancreatic acinar cell carcinoma after PD-1 blockade integrated treatment

**DOI:** 10.3389/fimmu.2025.1663514

**Published:** 2025-12-02

**Authors:** Xinyuan Bai, Di Yang, Jingjing Chen, Yaru Liu, Haochen Tang, Xiang Kong, Baorui Liu, Huizi Sha, Juan Du

**Affiliations:** 1 Department of Oncology, Nanjing Drum Tower Hospital, Affiliated Hospital of Medical School, Nanjing University & Clinical Cancer Institute of Nanjing University, Nanjing, China; 2 Department of Oncology, Nanjing Drum Tower Hospital, Clinical College of Nanjing Drum Tower Hospital, Nanjing University of Chinese Medicine, Nanjing, China; 3 Department of Oncology, Nanjing Drum Tower Hospital, Clinical College of Nanjing Drum Tower Hospital, Nanjing Medical University, Nanjing, China; 4 Department of Oncology, Nanjing Drum Tower Hospital, Clinical College of Nanjing Drum Tower Hospital, Jiangsu University, Nanjing, China

**Keywords:** pseudoprogression, pancreatic acinar cell carcinoma, PD-1 blockade, antiangiogenic therapy, integrated treatment, case report

## Abstract

Pancreatic Acinar Cell Carcinoma (PACC) is a rare subtype of pancreatic cancer, and its systematic treatment protocol is still pending for further exploration. There have been a few reports that PACC patients benefited from immunotherapy-integrated treatment. Here, we present a metastatic PACC patient who had new hepatic lesions but lowered serum AFP and CA19–9 after 2 cycles of integrated treatment that included chemotherapy, PD-1 blockade and antiangiogenic therapy, which was later confirmed to be pseudoprogression (PsP) by pathological diagnosis. So far as we know, this is the first reported PsP case of PACC. This case highlighted the value of biopsy in distinguishing PsP from authentic tumor progression of PACC.

## Introduction

Pancreatic Cancer is one of the most malignant cancers and ranks 6th among the cancer-related mortality (1), with Pancreatic Ductal Adenocarcinoma (PDAC) being its most common subtype. By contrast, originating from acinar cells, Pancreatic Acinar Cell Carcinoma (PACC) is a rare subtype and only accounts for 1~2% of overall pancreatic cancer cases (2). From a general view, the prognosis of PACC is better than PDAC but worse than pancreatic neuroendocrine tumor, with a rough median overall survival of 47 months (3). Although radical resection at localized or even oligometastatic stage has been confirmed to be correlated with improved survival of PACC (4, 5), its tendency of recurrence and metastasis renders it important to explore novel whole-body systematic treatment regimen. Interestingly, a limited number of recent cases have revealed the potential superiority of comprehensive therapy that includes chemotherapy, immunotherapy and antiangiogenic targeted therapy over chemotherapy alone in PACC treatment (6–8), which is believed to have synergistic effect on reconstituting tumor immune microenvironment by multiple means, which ultimately result in cytotoxic T lymphocyte infiltration in tumor microenvironment and enhanced therapeutic effect of immunotherapy (9).

In terms of solid tumors, pseudoprogression (PsP) refers to the phenomenon in which initial increase in tumor sizes occurs or new lesions emerge followed by a decrease in tumor burden (10), usually after Immune Checkpoint Inhibitor (ICI) treatment or other forms of immunotherapy. Despite controversy on its detailed definition, PsP is closely associated with elevated immunocompetent cell level and intensified inflammatory reaction in tumor microenvironment (11), which are potential indicators of therapeutic response and lengthened survivals (12, 13). Immunotherapy-related pseudoprogression is most common in Melanoma, followed by Renal Cell Carcinoma (RCC), Urothelial Carcinoma and Non-Small Cell Lung Carcinoma (NSCLC) (10). By comparison, ICIs are rarely applied in the treatment of pancreatic cancer owing to its disappointing survival outcomes in previous clinical trials (14, 15), which is largely attributed to its nature of immune desert. Thus, there is scarcely any cases of PsP in pancreatic cancer patients.

Here, we present a case of PACC patient who rapidly underwent hepatic metastasis shortly after radical resection. PsP of metastatic hepatic cancer was observed after 2 cycles of comprehensive treatment including Nab-paclitaxel and Gemcitabine chemotherapy, Penpulimab (a PD-1 blockade) and Anlotinib (an antiangiogenic targeted Tyrosine Kinase Inhibitor). This case highlights a clinical scenario where, despite radiologic tumor progression, patients receiving ICI-integrated therapy for PACC may exhibit improved serum tumor markers and quality of life. In such cases, biopsy remains the gold standard to distinguish true disease progression from PsP.

## Case presentation

**Figure 1:**
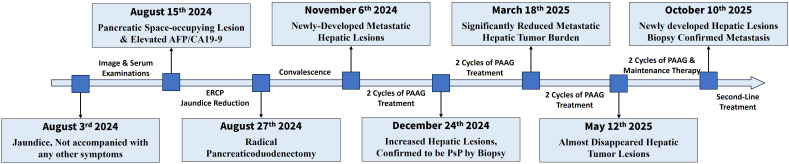
Overview of course of disease.

**Figure 2:**
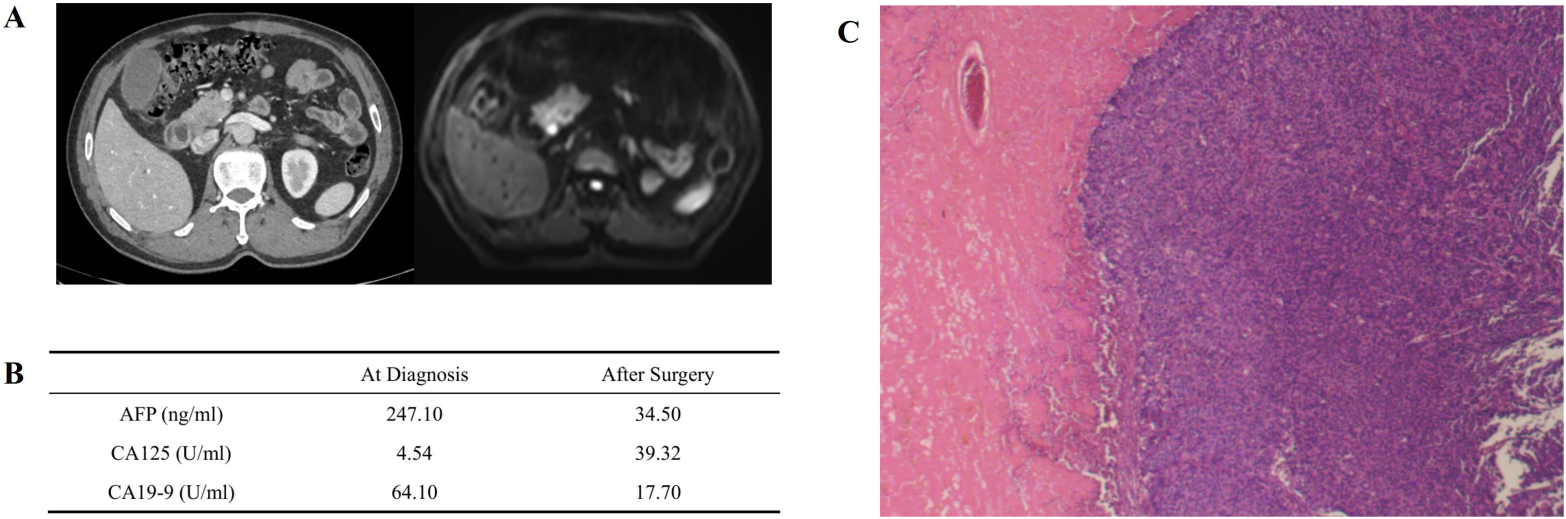
Primary lesion, preoperaitve/postoperative serum tumor marker and pathologic image. **(A)** Primary pancreatic lesion view under contrast CT (left) and contrast MR (right). An obvious lesion locates at head of pancreas can be found. **(B)** Serum tumor marker before and after surgery. AFP and CA19–9 decreased significantly after surgery, and slightly increased CA125 may be caused by surgery-associated ascitic fluid. **(C)** Pathological image of primary lesion, demonstrating poorly differentiated carcinoma with necrosis.

The overview of treatment process of the patient is depicted in **Figure 1**. A 61-year-old male patient came to our hospital for treatment in August, 2024 due to jaundice for 10 days, not accompanied with any other digestive symptoms. He was normally in good health, with no chronic diseases except stable-controlled hypertension and had no family history of cancer. An elevated level of serum Alpha Fetoprotein (AFP, 247.10ng/ml, **Figure 2B**) and Carbohydrate Antigen 19-9 (CA19-9, 64.10U/ml, **Figure 2B**) was detected, and imaging studies found a pancreatic lesion (**Figure 2A**). No metastatic sites were found. After his symptom of jaundice was controlled by stent implementation and medical treatment, the patient was evaluated as resectable pancreatic cancer by Multidisciplinary Team (MDT) discussion. After exclusion of contraindications, the patient underwent radical pancreaticoduodenectomy. Postoperative pathology (**Figure 2C**): poorly-differentiated acinic cell carcinoma, R0 margin, vascular invasion (-), perineural invasion (-), lymph node metastasis (3/11), pathological stage (pT_2_N_1_cM_0_, II_B_). Immunohistochemical: Cytokeratin (CK) (++), Ki67 (approximately 60%+), Synaptophysin (Syn) (+), Chromogranin A (CgA) (-), CD56(-), D2-40 (Vascular negative), CD31 (Vascular positive), Insulinoma-Associated Protein 1 (INSM1) (-), Somatostatin Receptor 2A (SSTR2A) (-), Alpha-Thalassemia/X-Linked Intellectual Disability Syndrome Gene (ATRX) (+), P53 (Positive at different intensity), Thyroid Transcription Factor-1 (TTF-1) (-), Trypsin (+++), B-Cell Lymphoma/Leukemia 10 (Bcl10) (-), CD10 (-), β-catenin (membrane positive), AFP (partially positive). The patient developed postoperative complications but soon recovered from them shortly after radical surgery, which included pancreatic fistula, intra-abdominal infection and COVID-19 infection.

**Figure 3:**
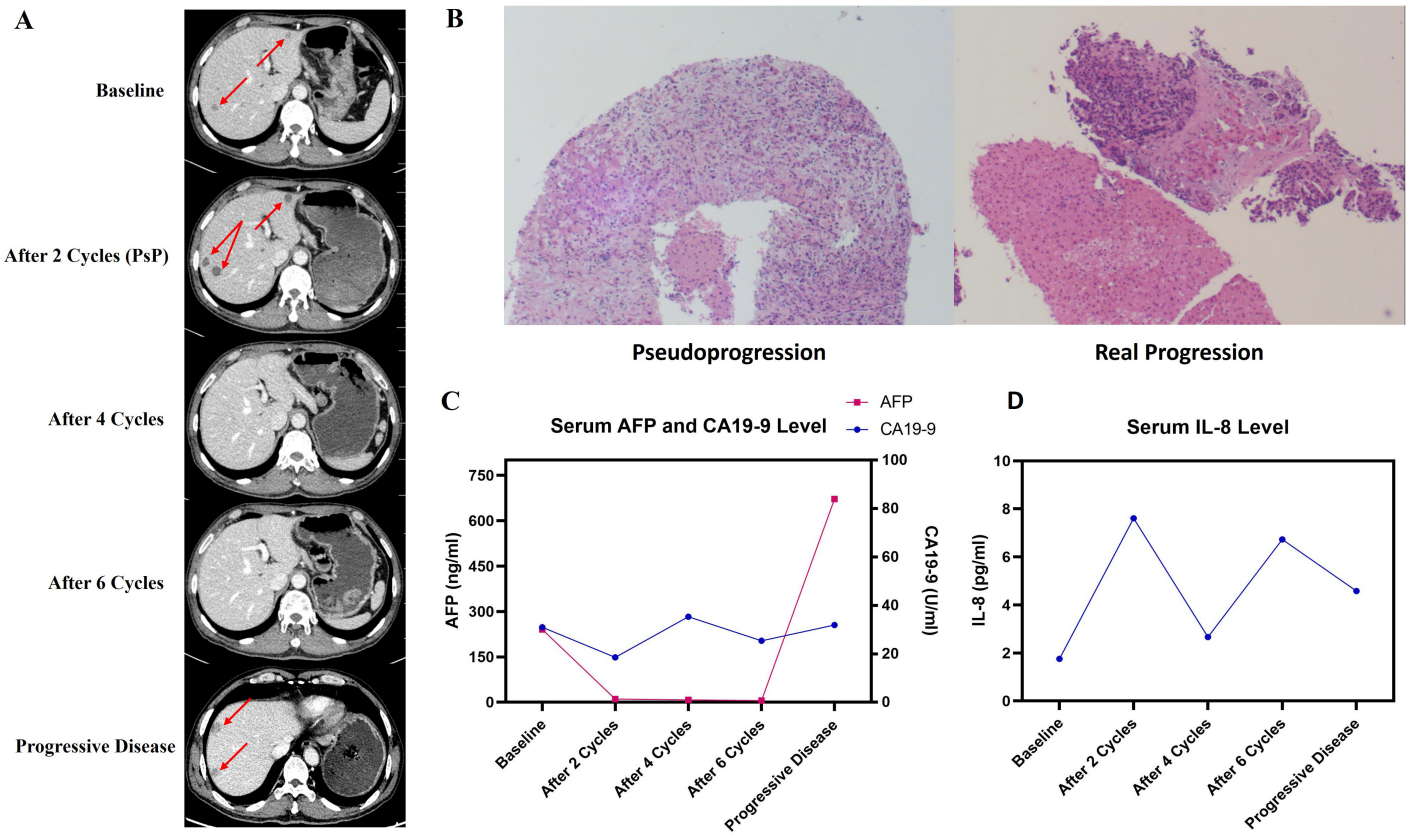
Efficacy of PAAG regimen by follow-up. **(A)** Outcome of radiologic follow-up by contrast CT. **(B)** Pathological images of PsP (left) and real progression (right) by hepatic biopsy. **(C)** Serum AFP and CA19–9 level at different follow-up points. **(D)** Serum IL-8 level at different follow-up points.

Because of the patient’s diminished fitness and personal reluctancy to further adjuvant therapy, the first postsurgical radiomic follow-up was not performed until November 6^th^, 2024. Unfortunately, new hepatic lesions (**Figure 3A**) and elevated serum AFP & CA19-9 (**Figure 3C**) were observed, indicating that rapid distant metastasis occurred shortly after surgery. NGS and PD-L1 expression were implemented in seek for potential benefit from immunotherapy or targeted therapy, result: Tumor Mutation Burden (TMB): 1.42Muts/Mb, Microsatellite Stability (MSS), KRAS wild type, BCL6/RPS6KA3/GATA6/TCF7L2/GNAS/FANCC/RICTOR mutation, Tumor Proportion Score (TPS): 3%, Combined Positive Score (CPS): 5%.

Two senior oncologists systematically reviewed the patient’s overall course of disease. Given the previous PACC cases benefiting from comprehensive treatment, the patient was advised to participate in the Investigator Initiated Trial *Penpulimab Combined With Anlotinib and Nab-paclitaxel Plus Gemcitabine as First-line Treatment for Advanced Metastatic Pancreatic Cancer (RCT-PAAG)* (NCT06051851) and was successfully enrolled in the experimental group. Starting on November 8^th^, the patient received treatment regimen of Penpulimab (a PD-1 blockade), Anlotinib (an antiangiogenic targeted drug), Nab-paclitaxel and Gemcitabine (PAAG) as first-line regimen. Follow-up were carried out every 2 cycles, containing contrast-enhanced CT, serum tumor marker and serum cytokine. Intriguingly, after 2 cycles of treatment, newly-developed and increased hepatic lesions were found by contrast CT, but his serum AFP and CA19–9 decreased to the novel range. The patient also stated that his abdominal and digestive symptoms has relieved after 2 cycles of PAAG therapy, which was contradictive to his radiomic result. The patient complained of rash and itching after the first cycle of therapy, and these symptoms soon vanished after treated with Loratadine tablets.

Another MDT discussion considered that possibility of PsP should not be excluded. To clarify the nature of new hepatic lesions, the patient underwent hepatic biopsy under color Doppler ultrasound guidance. Histopathology: focal hepatocyte necrosis accompanied by lymphocyte infiltration and fibrocyte reaction, no evidence of malignant tumor (**Figure 3B**). Pathological outcomes confirmed PsP of hepatic lesions, which are response to PD-1 Inhibitors. Therefore, the patient continued to receive PAAG treatment for another 2 cycles. In subsequent follow-ups, vanished lesions and decreased AFP further confirmed the nature of PsP.

By June 17^th^, 2025, the patient has finished 8 cycles of PAAG regimen and was in good health. According to the protocol of the trial, he then entered the stage of chemotherapy-free maintenance therapy (i.e. Penpulimab plus Anlotinib only). During the whole process, the patient did not exhibit treatment-related adverse events except tolerable anemia and neurotoxicity, and no severe adverse event was reported. His scores of Eastern Cooperative Oncology Group Performance Status (ECOG-PS) and Nutritional Risk Screening 2002 (NRS-2002) remained at 0 throughout the PAAG treatment, and always had complete self-care ability. On October 10^th^ 2025, however, newly-developed hepatic lesions (**Figure 3A**) and significantly elevated AFP (**Figure 3C**) were observed. The patient insisted on another biopsy to exclude the probability of another PsP, but following pathological outcomes confirmed its nature of metastatic tumor (**Figure 3B**). The second-line regimen was Liposomal Irinotecan plus Fluorouracil and Calcium Folinate, which referenced the standard second-line regimen of PDAC. By last follow-up conducted on October 31^st^, 2025, the patient was in good condition, with a Progression-Free Survival of 11.1 months and is still undergoing second-line treatment.

## Discussion

This article describes one rare instance of pseudoprogression (PsP) in a patient with metastatic Pancreatic Acinar Cell Carcinoma (mPACC) following first-line treatment regimen of Penpulimab (a PD-1 blockade), Anlotinib (an antiangiogenic targeted drug), Nab-Paclitaxel and Gemcitabine chemotherapy (PAAG). His radiomic follow-up after two cycles revealed new hepatic lesions, which met RECIST criteria for disease progression but were eventually confirmed to be PsP through pathological biopsy and subsequent radiomic follow-ups. To the best of our knowledge, this is the first reported PACC case of immunotherapy-induced PsP. Given that PACC constitutes <2% of pancreatic malignancies (2) and reports of PsPs in this subtype are exceedingly scarce, this case underscores a critical diagnostic pitfall and therapeutic opportunity.

PsP following Immune Checkpoint Inhibitor (ICI) treatment is largely attributed to transient lymphocyte-inflammatory infiltration, which radiologically resembles tumor progression (i.e. increased or newly-merged lesions). While PsP is well-documented in melanoma or NSCLC, its occurrence in PACC is novel. In our case, the combination of chemotherapy, antiangiogenic targeted therapy and PD-1 blockade together successfully transformed one metastatic hepatic site into ‘tumor immune-graveyard’, which can be inferred from immunohistochemical outcomes: The strong positivity of CK and negativity of Trypsin is are indicators of tumor exocrine impotency, suggesting that metabolic-active tumor cells of this lesion have been eradicated after combined PAAG treatment. Besides, scattered positive expression of CD4+, CD8+, CD20+ and MPO are signals of CTL, Th cell, lymphocyte B cell and neutrophil cell infiltration triggered by immunological response, respectively. This case suggests that even in “cold” tumors like PACC, robust immune responses can manifest as PsP, hinting at latent immunogenicity.

Distinguishing PsP from real progressive disease in PACC is challenging yet critical, since such confusions would result in premature abortion of effective treating measures and worsened prognosis. As for melanoma or NSCLC where PsP occurs relatively more frequently, reported methods of differentiation includes radiomic features and peripheral blood results (16, 17). For instance, declined IL-8 or cell-free DNA (ctDNA) levels are latent biomarkers of ICI-treatment responses (18, 19), and thus may function as convenient approaches for distinction. In this case, however, PsP were observed with a transiently increased level of IL-8 (**Figure 3D**). We predict that this intriguing phenomenon is because that chemotherapy, PD-1 blockade and antiangiogenic targeted therapy together induced tumor cell necrosis, which triggered release of tumor neoantigen and subsequent local inflammation (20), where elevated IL-8 and neutrophil infiltration serve as responders (21). Also, in this case, serum AFP levels were more parallel to tumor burdens than CA19-9 (**Figure 3C**), and we speculate that serum AFP plays a significant role in efficacy monitoring of PACC if positive. Anyway, biopsy, as the gold standard, should always be considered when confronting such perplexing situation, especially for “cold” tumors such as pancreatic or biliary tract cancer where PsP scarcely occurs.

In this case, the patient benefitted from the combination treatment of chemotherapy, immunotherapy and targeted therapy. Similar strategies also revealed potentially improved efficacy over chemotherapy alone in late-stage non-small-cell lung carcinoma, gastric cancer and colorectal cancer (22–26). The synergistic effect of immune checkpoint inhibitor and antiangiogenic targeted therapy can be viewed from several different perspectives. Firstly, antiangiogenic therapy is capable of normalizing abnormal tumor vessel. As a result, the infiltration of immunologic effector cell (NK cell, cytotoxic T cell, etc.) and delivery of drugs (including ICIs) are promoted (27–29). Normalized tumor vessel also transformed the oxygen-deficient tumor microenvironment, which inhibits immune escape so as to prevent ICI drug resistance (30, 31). In addition, VEGF has been confirmed to abrogate dendritic cell activation (32) and enhance expression of PD-1 and other inhibitory checkpoints involved in CD8^+^T cell exhaustion (33), and antiangiogenic drugs targeting VEGF remove the inhibition of immune killing (34). In this sense, the integrated therapy of ICI and antiangiogenic drug is worth attempting for “cold” tumors such as pancreatic or biliary cancer based on standard chemotherapy regimens. Meanwhile, the additional risk by immunotherapy and antiangiogenic therapy should not be overlooked.

In conclusion, we reported an immunotherapy-related pseudoprogression case of metastatic PACC patient. This case stressed the importance of comprehensive outlook when evaluating efficacy of immunotherapy for pancreatic cancer patients, especially when increased or newly-developed lesions come along with decreased tumor marker and improved quality of life. In addition, biopsy, as the gold standard, should always be implemented when possible and necessary.

## Data Availability

The raw data supporting the conclusions of this article will be made available by the authors, without undue reservation.
