# Supplementary material for: Case Report: Pseudoprogression of pancreatic acinar cell carcinoma after PD-1 blockade integrated treatment
Source: Front Immunol. 2025 Dec 2;16:1663514. doi: 10.3389/fimmu.2025.1663514 (PMC12705604; doi:10.3389/fimmu.2025.1663514)
Supplement: Supplementary file 1 [file Table1.docx]

***Supplementary Materials For***

**Case Report: Pseudoprogression of Pancreatic Acinar Cell Carcinoma after PD-1 Blockade Integrated Treatment**

Xinyuan Bai^1,2^, Di Yang^1,3^, Jingjing Chen^1,2^, Yaru Liu^1,2^, Haochen Tang^1,3^, Xiang Kong^1,4^, Baorui Liu^1✉^, Huizi Sha^1✉^ and Juan Du^1,2,3,4✉^

**1** Department of Oncology, Nanjing Drum Tower Hospital, Affiliated Hospital of Medical School, Nanjing University & Clinical Cancer Institute of Nanjing University,

Nanjing, China

**2** Clinical College of Nanjing Drum Tower Hospital, Nanjing University of Chinese Medicine, Nanjing, China

**3** The Comprehensive Cancer Center, Nanjing Drum Tower Hospital, Clinical College of Nanjing Medical University, Nanjing, China

**4** Drum Tower Clinical Medical College of Jiangsu University, Zhenjiang, China

**Supplementary Table 1. Peripheral Blood Cytokine Level at Different Period (pg/ml)**

| _Cytokine_\^Period^ | **Baseline** | **After 2 Cycles** | **After 4 Cycles** | **After 6 Cycles** | **Progressive Disease** |
| --- | --- | --- | --- | --- | --- |
| **IL-1β** | 0.03 | 0.11 | 0.13 | 0.39 | 1.22 |
| **IL-2** | 0.18 | 0.19 | 0.31 | 0.23 | 0.81 |
| **IL-4** | 1.50 | 1.29 | 2.60 | 1.39 | 1.20 |
| **IL-5** | 0.53 | 0.50 | 0.89 | 0.66 | 1.15 |
| **IL-6** | 5.69 | 8.39 | 3.60 | 4.04 | 24.67 |
| **IL-8** | 1.75 | 7.61 | 2.66 | 6.73 | 4.58 |
| **IL-10** | 1.17 | 1.54 | 1.62 | 1.54 | 6.45 |
| **IL-12p70** | 0.53 | 0.79 | 0.79 | 1.18 | 1.35 |
| **IL-17** | 0.31 | 0.38 | 0.79 | 0.51 | 2.10 |
| **IFN-α** | 0.25 | 0.30 | 0.38 | 0.61 | 2.90 |
| **IFN-γ** | 1.34 | 1.59 | 1.88 | 3.34 | 2.79 |
| **TNF-α** | 1.54 | 1.63 | 1.78 | 0.79 | 2.67 |

**Supplementary Table 2. Peripheral Blood Tumor Marker at Different Period**

| _Marker_\^Period^ | **Baseline** | **After 2 Cycles** | **After 4 Cycles** | **After 6 Cycles** | **Progressive Disease** |
| --- | --- | --- | --- | --- | --- |
| **AFP (ng/ml)** | 240.50 | 10.30 | 7.70 | 5.40 | 671.50 |
| **CEA (ng/ml)** | 1.07 | 0.91 | 1.08 | 1.17 | 1.44 |
| **CA125 (U/ml)** | 9.21 | 4.41 | 5.22 | 5.00 | 5.60 |
| **CA19-9 (U/ml)** | 31.00 | 18.50 | 35.30 | 25.40 | 31.90 |
| **CA72-4 (U/ml)** | 1.08 | 3.11 | 0.97 | 1.10 | 1.55 |
| **CA242 (IU/ml)** | 9.31 | 5.82 | 9.69 | 6.74 | 4.26 |
